# Ultrasonographic and macroscopic study of pregnancy in golden hamster

**DOI:** 10.1186/s42826-022-00112-9

**Published:** 2022-01-31

**Authors:** Mohammad Amin Keshavarz, Asghar Mogheiseh, Mohammad Saeed Ahrari-Khafi, Reza Mahboobi

**Affiliations:** 1 Department of Clinical Sciences, School of Veterinary Medicine, Shiraz University, P.O. Box 7144169155, Shiraz, Fars Iran; 2 Central Laboratory, School of Veterinary Medicine, Shiraz University, Shiraz, Fars Iran

**Keywords:** Gestational age, Hamster, Synchronization, Ultrasound

## Abstract

**Background:**

Hamster is widely used as an experimental model in the study of reproductive system. However, pregnancy diagnosis and aging always have been a challenge. ultrasonography have been used in diagnosis of pregnancy in some small laboratory animals, such as rabbits, rats, and mice. Current study describes use of trans-abdominal ultrasonography for pregnancy diagnosis and fetal age estimation in golden hamster. Furthermore, a macroscopic examination was performed to evaluate the embryonic vesicle diameter, crown-rump length, and fetal head diameter. Ten adult female golden hamsters were selected and maintained under controlled light conditions (14 h light/10 h darkness). The estrous cycle was synchronized using eCG and hCG. During estrous (18 h after hCG injection), the hamsters were naturally mated. After seven days of mating, the hamsters were examined daily for pregnancy diagnosis and aging with an ultrasound scanner equipped with an 8.5-MHZ linear probe. On each day of the experiment, at least one of the pregnant hamsters was euthanized and dissected for macroscopic fetal measurements using a digital caliper.

**Results:**

The gestational sac and crown-rump length were identified and measured by ultrasonographicly on day 7 of pregnancy and head could be visible after day 10 of gestation. Statistical analysis revealed that the ultrasound estimation of gestational age was significantly correlated with the actual age of the fetus (r = 0.98; *p* < 0.05).

**Conclusions:**

Real-time ultrasound can be used for the diagnosis of pregnancy and estimation of fetal age in golden hamster from day 7 of gestation.

## Background

Ultrasound has been widely used to assess human pregnancy, but it has not been very common in studies of pregnancy in animals [1]. Up until recently, the use of ultrasound has been restricted to non-rodent species [2]. Abdominal palpation, as a method of pregnancy diagnosis, can only be used from mid-to-late pregnancy in laboratory animals [3]. Hamster is a polytocous animal with a short length of pregnancy period and is widely used as an experimental model in the study of human reproduction system [4]. Hamster is also used in reproductive toxicology studies that require early detection of pregnancy and identification of fetal growth abnormalities. Pregnancy ultrasound examination is common in domestic animals (e.g., horse, cattle, goat, sheep, swine, dog, and cat), laboratory animals (e.g., ferret, guinea-pig, rabbit, and rat), exotic ungulates (e.g., deer, antelopes, anoa, banteng, giraffe, camelids, and rhinoceros), and zoo and wild animals (e.g., primates, dolphin, shark, and opossum) [5]. Ultrasound examination is used experimentally in some small laboratory animals, such as rabbits [6], rats [7], and agoutis [8]. Some studies have shown the effective use of ultrasound backscatter microscopy in examining the fetal development in mice, in particular the development of the embryonic eye, heart, and central nervous system [9].

Male hamster reaches maturity at 6–8 weeks of age and is fertile from 12 weeks of age. Female hamster usually reaches maturity at 5–6 weeks of age and is able to breed from 6–7 weeks of age. Female hamsters kept in the same group tend to fight; therefore, a 1:1 mating ratio is the most effective option [10]. Single mating requires an accurate diagnosis of estrus. The female can then be placed in a male cage for mating. The estrous cycle lasts about four days. Ovulation occurs 8 to 10 h after estrus. Increased vaginal discharge, especially on the third day, is a sign of estrus. The gestation period is 21 days [11]. The anatomy of reproductive tract has unique features in hamsters in comparison to other rodent species. The cranial part of cervix composes of two canals and extends to duplex uterus. The vagina has two lateral pouches that must be distinguished from vaginal canal for sampling and staging of estrous cycle [11].

Different doses and timings have been evaluated for estrous synchronization and superovulation in hamster. PMSG (eCG) and hCG are the common hormones in these protocols. Injection of 5 IU of eCG and hCG and mating 18 h after hCG provided appropriate results in superovulated golden hamsters [12].

To the best of our knowledge, ultrasound has not been used to diagnose pregnancy in hamster. It seems that pregnancy diagnosis and aging is difficult in hamster due to some specific stress responses during restraint. This study aimed to describe the use of trans-abdominal ultrasonography for pregnancy diagnosis and fetal age estimation in golden hamster. Furthermore, a macroscopic examination was performed for the measurement of embryonic vesicle diameter, crown-rump length, and the diameter of the fetal head. The accuracy of ultrasound in estimating the gestational age was evaluated by comparing its estimation with macroscopic measurements.

## Results

**Fig. 1:**
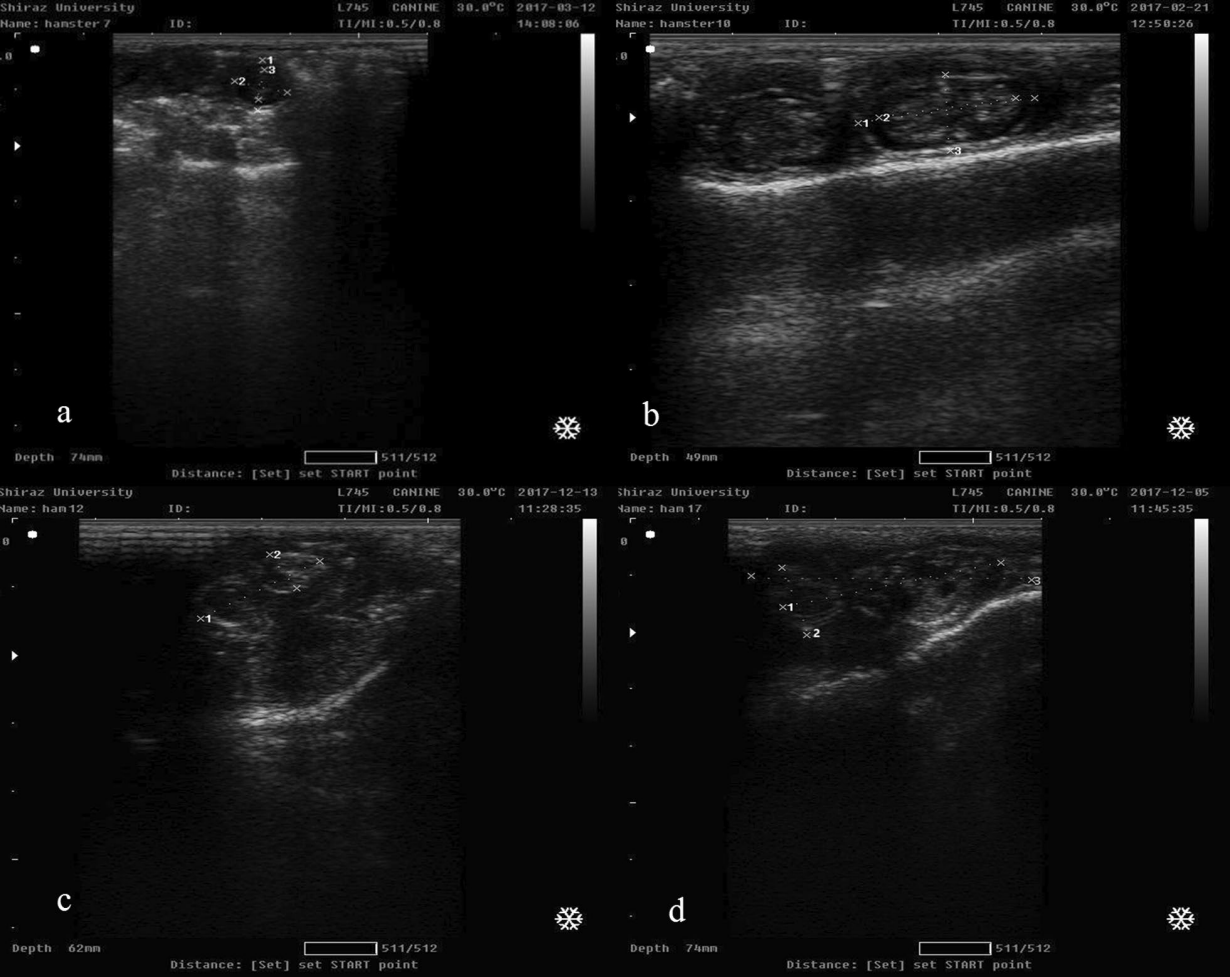
The ultrasound images on days 7 (**a**), 10 (**b**), 12 (**c**), and 17 (**d**) of pregnancy in golden hamsters. Crown-rump length (CRL), gestational sac diameter (GSD), and head diameter (HD) were used to estimate the gestational age of golden hamsters

**Fig. 2:**
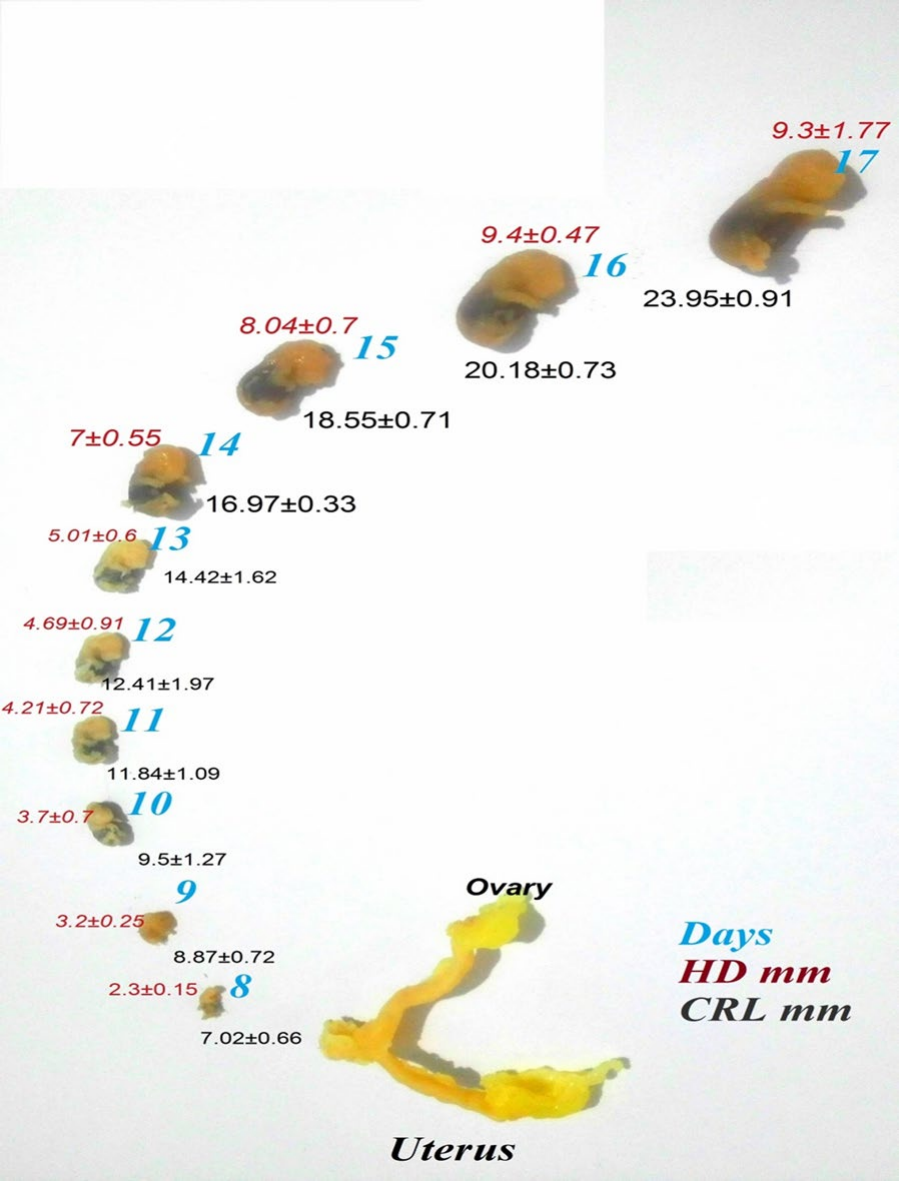
The macroscopic evaluation of the fetus from day 7 to day 17 of pregnancy in golden hamsters. The indices used included crown-rump length (CRL) and head diameter (HD)

In the ultrasound examination, gestational sac diameter was the earliest diagnostic index of pregnancy and could not be reliably measured until day 7 of gestation (Fig. 1). The mean diameter of the embryonic vesicle was 11.2 ± 0.0 mm on day 7 and 25.2 ± 2.6 mm on day 17. Head diameter could be measured from day 10 of pregnancy. The average head diameter was 4.15 ± 0.46 mm on day 7 and 10.21 ± 1.58 mm on day 17 of pregnancy. The crown-rump length was 7 ± 0.0 mm on day 7 and 24.55 ± 1.2 mm on day 17 of pregnancy (Table 1; Fig. 2).

**Table 1:** Ultrasonographic (US) and macroscopic (Mac) examination of fetuses and gestational sacs during day 7–17 of pregnancy in golden hamsters

Day	Number of fetus (Mac)	CRL (mm; Mac)	HD (mm; Mac)	Number of fetus (US)	GSD (mm; US)	CRL (mm; US)	HD (mm; US)
7	4	6.19 ± 0.48	2.1 ± 0.31	2	11.2 ± 0.0	7 ± 0.0	
8	5	7.02 ± 0.66	2.3 ± 0.15	2	11.5 ± 0.66	7.86 ± 0.31	
9	5	8.87 ± 0.72	3.2 ± 0.25	3	12.4 ± 1.01	9.35 ± 0.64	
10	5	9.5 ± 1.27	3.7 ± 0.7	2	13.67 ± 0.17	10.21 ± 1.18	4.15 ± 0.46
11	6	11.84 ± 1.09	4.21 ± 0.72	2	15.84 ± 0.24	12.4 ± 1.24	4.73 ± 0.4
12	6	12.41 ± 1.97	4.69 ± 0.91	3	16.66 ± 0.68	13.8 ± 3.8	5.2 ± 0.6
13	6	14.42 ± 1.62	5.01 ± 0.6	3	17.2 ± 0.0	15.1 ± 0.0	6 ± 0.0
14	5	16.97 ± 0.33	7 ± 0.55	4	18.89 ± 0.29	16.6 ± 1.62	7.59 ± 1.01
15	5	18.55 ± 0.71	8.04 ± 0.7	2	20.1 ± 0.0	18.88 ± 0.0	9 ± 0.0
16	5	20.18 ± 0.73	9.4 ± 0.47	3	22.44 ± 0.62	20.8 ± 0.34	10.2 ± 0.36
17	6	23.95 ± 0.91	9.3 ± 1.77	2	25.2 ± 2.6	24.55 ± 1.02	10.21 ± 1.58

*CRL* crown-rump length, *HD* head diameter, *GSD* gestation sac diameter

In the macroscopic measurements of the fetus, head diameter could be measured from day 7 of pregnancy. The average head diameter was 2.1 ± 0.31 mm on day 7 and 9.3 ± 1.77 mm on day 17 of pregnancy. The crown-rump length was 6.19 ± 0.48 mm on day 7 and 23.95 ± 0.91 mm on day 17 of pregnancy (Table 1). The total number of fetuses removed from the uteri was 58, and the total number of fetuses detected on ultrasound examination was 28.

**Fig. 3:**
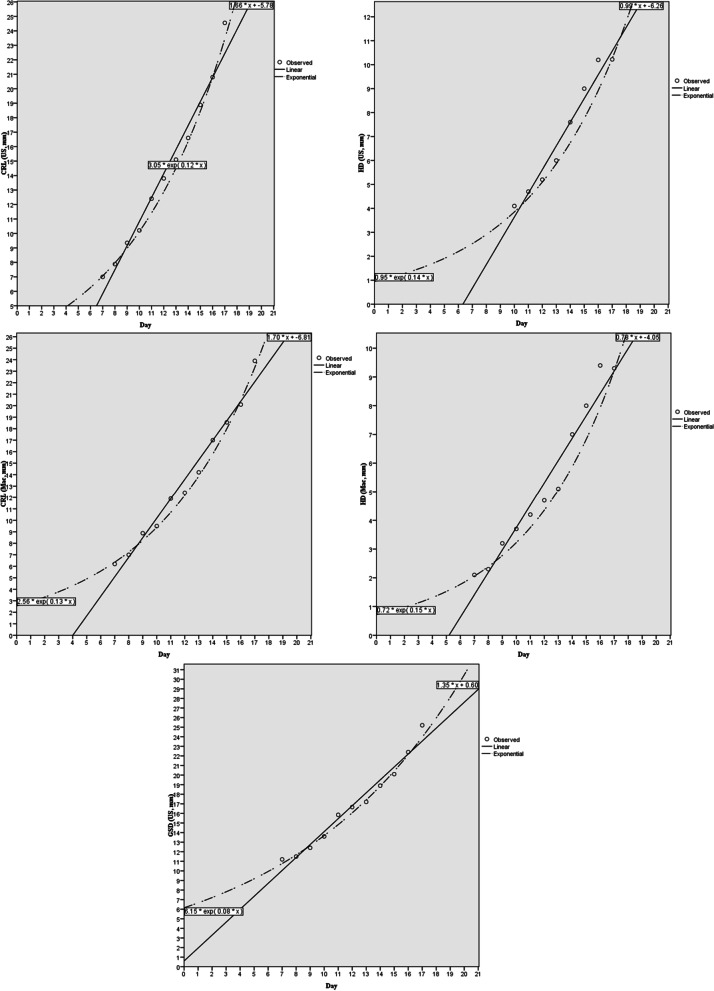
Correlation between days of pregnancy and ultrasonographic (us) and macroscopic (mac) measurements was presented for fetus and gestation sac of pregnant golden hamster. CRL: crown-rump length; HD: head diameter; GSD: gestation sac diameter

The positive and significant correlation was found between days of pregnancy and ultrasound measuring of CRL, HD and GSD and between days of pregnancy and macroscopic measuring of CRL and HD (Fig. 3). Also, there was significant correlation between ultrasound and macroscopic measuring of CRL and HD.

## Discussion

In this study, early pregnancy detection was possible in golden hamster from day 7 of gestation by trans-abdominal ultrasound examination. It seems that GSD, CRL, and HD are good indices for the estimation of gestational age in golden hamster. In pregnant laboratory animals, abdominal palpation, as a method of pregnancy diagnosis, is possible from mid-to-late pregnancy. For example, in rabbits, the gestation period is 30 days, and pregnancy diagnosis can be performed by abdominal palpation from day 12 of pregnancy. Ultrasound pregnancy diagnosis is much more accurate than abdominal palpation [3].

Furthermore, tumors and infections may be mistaken with pregnancy in abdominal palpation. Nowadays, due to advances in high-frequency ultrasound imaging, it is possible to obtain accurate images of internal organs, such as the heart [13] and kidney [14] of rodents, such as mice and rats. Linear probes of 7.5–12 MHz have been used for ultrasound examination of pregnancy in laboratory animals. In a study on a group of mice, it was found that linear probes produced higher-resolution images than curved probes did at higher frequencies [15].

The results of the present study showed that prenatal fetal ultrasound evaluations were largely consistent with the macroscopic results. The macroscopic measurements obtained immediately after the ultrasound examination. It can be stated that gestational age can be estimated in golden hamster using ultrasound data from day 7 of pregnancy. In a study on mice, a10 MHz linear transducer was used, and the embryo and gestational sac could be clearly distinguished at day 8.5 [15]. In a different study on rats, the uteri of anesthetized Wistar rats were examined between 9 and 16 days post-coitum by trans-abdominal real-time ultrasound using a 12-MHz linear transducer. Color and pulsed-wave Doppler ultrasonography were used to measure the embryonic heart rate. The embryonic vesicles were detected with a 25% false-negative diagnosis rate on day 9, 8% on day 10, and 0% thereafter. Crown-rump length and heart rate were detected from day 12. Ultrasonographic evidence of pregnancy could be found in hamster by day 9 post-coitum. The diagnosis of pregnancy was confirmed by day 12 when the embryonic heartbeat was detected. Embryonic characteristics were ultrasonographically detectable between days 9 and 16 [16]. In this study, we found that the diagnostic values of ultrasound were lower than the macroscopic values. This discrepancy in values may be due to the curved and special shape of the fetus in the gestational sac. However, it is clear that during pregnancy, as the fetus grows so does the average embryonic vesicle size, head diameter, and crown-rump length. In this study, we observed that the fetal growth rate increased more in the last few days of pregnancy compared to the early and mid-pregnancy. If the pregnancy period from day 9 to the end is divided into three periods of approximately three days, the average CRL size was 10.07 ± 1.02 mm in the first three days, 14.6 ± 1.3 mm in the second three days, and 20.89 ± 0.78 mm in the third three days (Table 1). These data show that the fetus grew much more rapidly in late pregnancy. Thus, it could be suggested that fetal growth was faster in the last days of pregnancy in golden hamster. Many studies have been conducted on the effects of cancer or the use of drugs during pregnancy [17]. Congenital heart disease is the most common non-infectious cause of death at birth. Advanced methods such as ultrasound imaging allow live imaging of abnormal hearts in embryos affected by heart failure [18]. Such advanced methods provide us with valuable data on the development of the fetus during diseases. These data allow researchers to investigate any possible association between drugs, toxins, and vaccines on the one hand, and the risk of miscarriage, or even their effects on fetal development during pregnancy on the other hand [14]. Therefore, more studies are needed to evaluate pregnancy and fetal growth and viability in different species.

## Conclusions

In this study, early pregnancy diagnosis was possible from day 7 of gestation by transabdominal ultrasound examination in golden hamsters. It seems that GSD, CRL, and HD are good indices for the estimation of gestational age. It is suggested that future studies investigate the effects of ultrasound on the development of fetal internal organs. Studies on the growth of each organ during pregnancy can be helpful in enhancing our understanding of pregnancy, genetic diseases, and the effects of drugs on the fetus. Finally, it is recommended that the weight of fetuses be estimated using fetal biometric parameters, such as biparietal diameter and fetal abdominal circumference.

## Methods

Experimental protocols were performed in accordance with the Iranian animal ethics framework under the supervision of the Iranian Society for the Prevention of Cruelty to Animals and Shiraz University Research Council (IACUC no: 4687/63).

### Animals

Ten adult hamsters were selected and housed in an individually ventilated caging system under controlled temperature (21–23 °C) and light (14 h light/10 h darkness) conditions. They received a standard laboratory rodent diet and water ad libitum.

### Estrous synchronization, ultrasound examination and macroscopic evaluation

**Fig. 4:**
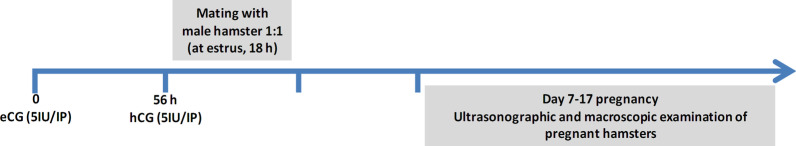
The study timeline, including superovulation, mating, and ultrasound and macroscopic examination of pregnant golden hamsters

The estrous cycle is repeated every 4 days in female hamster [10]. Five units of eCG were injected intraperitoneally to induce superovulation. Ovulation was induced by injecting 5 units of hCG after 54 h. Estrus is confirmed by vaginal discharge, which occurs a few hours after the hCG injection [12]. In hamster, vaginal viscous and milky white discharge with a distinct odor is observed in the morning after ovulation. The cornified epithelial cells are plentiful in the vaginal smear. Therefore, the vaginal discharge of female hamster was checked prior to mating to ensure that they had a normal estrous cycle. To determine the time of estrus, vaginal lavage was performed by the injection of 0.1 cc normal saline into the vagina and collected by a pipette. When inserting the pipette into and flushing the vagina, care was taken not to insert the pipette too deep so as to avoid cervical stimulation. Vaginal smears were observed under a light microscope at × 40 magnification to identify cells and × 10 magnification to determine the percentage of cells [19]. As soon as the hCG injection was administered and the time of estrus was determined, females and males were placed together in a 1:1 mating ratio for 18 h. The day of sperm detection in vaginal smears was considered as day zero of pregnancy. Transabdominal ultrasound examination was carried out before and after mating to day 17 of pregnancy with an 8.5-MHZ transducer (Sonoscape A6, china) based on the dimensions and echogenicity of the embryonic vesicle, head diameter and crown-rump length (Fig. 4). The hamsters were manually restrained in dorsal recumbence. Anesthesia was not used during the ultrasound examination to avoid any adverse effects on pregnancy. In each day of pregnancy diagnosis, one of the hamsters was anesthetized by Ketamine-Xylazine after the ultrasound examination. Then, cervical dislocation was performed during deep anesthesia [20]. The uterus was evacuated to access the embryos. The embryonic vesicle diameter, head diameter and crown-rump length were measured by a digital caliper.

### Statistical analysis

Statistical analysis of the data was performed using SPSS software and Pearson correlation test. Data are presented as mean ± SD. *P*-values less than 0.05 were considered significant.

## Data Availability

The dataset supporting the conclusions of this article is included within the article.

## Abbreviations

hCG: Human chorionic gonadotropin
eCG: Equine chorionic gonadotropin
CRL: Crown-rump length
HD: Head diameter
GSD: Gestation sac diameter
